# Studies on the in vitro and in vivo metabolism of the synthetic opioids U-51754, U-47931E, and methoxyacetylfentanyl using hyphenated high-resolution mass spectrometry

**DOI:** 10.1038/s41598-019-50196-y

**Published:** 2019-09-24

**Authors:** Frederike Nordmeier, Lilian H. J. Richter, Peter H. Schmidt, Nadine Schaefer, Markus R. Meyer

**Affiliations:** 1 Institute of Legal Medicine, Saarland University, 66421 Homburg, Germany; 2 Department of Experimental and Clinical Toxicology, Institute of Experimental and Clinical Pharmacology and Toxicology, Center for Molecular Signaling (PZMS), Saarland University, 66421 Homburg, Germany

**Keywords:** Mass spectrometry, Population screening, Bioanalytical chemistry

## Abstract

New Synthetic Opioids (NSOs) are one class of New Psychoactive Substances (NPS) enjoying increasing popularity in Europe. Data on their toxicological or metabolic properties have not yet been published for most of them. In this context, the metabolic fate of three NSOs, namely, trans-3,4-dichloro-*N*-[2-(dimethylamino)cyclohexyl]-*N*-methyl-benzenacetamide (U-51754), trans-4-bromo-*N*-[2-(dimethylamino)cyclohexyl]-*N*-methyl-benzamide (U-47931E), and 2-methoxy-*N*-phenyl-*N*-[1-(2-phenylethyl)piperidin-4-yl] acetamide (methoxyacetylfentanyl), was elucidated by liquid chromatography high-resolution mass spectrometry after pooled human S9 fraction (phS9) incubations and in rat urine after oral administration. The following major reactions were observed: demethylation of the amine moiety for U-51754 and U-47931E, *N*-hydroxylation of the hexyl ring, and combinations thereof. *N*-dealkylation, *O*-demethylation, and hydroxylation at the alkyl part for methoxyacetylfentanyl. Except for U-47931E, parent compounds could only be found in trace amounts in rat urine. Therefore, urinary markers should preferably be metabolites, namely, the *N*-demethyl-hydroxy and the hydroxy metabolite for U-51754, the *N*-demethylated metabolite for U-47931E, and the *N*-dealkylated metabolite as well as the *O*-demethylated one for methoxyacetylfentanyl. In general, metabolite formation was comparable *in vitro* and *in vivo*, but fewer metabolites, particularly those after multiple reaction steps and phase II conjugates, were found in phS9. These results were consistent with those of comparable compounds obtained from human liver microsomes, human hepatocytes, and/or human case studies.

## Introduction

There has been a rapid increase in New Psychoactive Substances (NPS) over the last decade. Many of them are not yet controlled and predominantly sold via the Internet as ‘legal’ replacements for drugs already subject to controls, such as heroin, cannabis or cocaine. New Synthetic Opioids (NSOs) are one class of NPS, mostly consisting of analogues of the highly potent analgesic fentanyl exploiting the fentanyl phenylpiperidine structure^1^. There are also other non-fentanyl synthetic opioids that share neither the fentanyl-like nor the classical morphine-like chemical structure. In Europe, their availability and popularity has grown continuously over recent years^2^. NSOs include compounds that were produced by pharmaceutical companies looking for new pharmaceutics, but were never registered for medical use due to strong side effects or insufficient action. Furthermore, NSOs are illegally synthesised in clandestine laboratories in China through modification of molecule residues^3–6^. Multiple cases of classical opioid toxidrome and several overdose deaths in Europe related to NSOs have been reported in recent years^3,7–10^. On the one hand, users are often not aware of consuming these compounds, because batches might be sold as heroin, other illicit opioids or even as counterfeit pain killers^3,11^. On the other, only little is known and published about the toxicology of these substances^12^.

Two non-fentanyl-related compounds discussed in drug user forums and available for sale on the Internet are trans-3,4-dichloro-*N*-[2-(dimethylamino)cyclohexyl]-*N*-methyl-benzenacetamide (U-51754) and trans-4-bromo-*N*-[2-(dimethylamino)cyclohexyl]-*N*-methyl-benzamide (U-47931E, bromadoline). Both are structurally related to 3,4-dichloro-*N*-[(1 R,2 R)-2-(dimethylamino)cyclohexyl]-*N*-methylbenzamide, commonly known as U-47700, and were synthesised by the Upjohn Company in an attempt to produce non-addicting analgesics as potent as morphine. Oral doses for U-51754 range from 12 to 25 mg and for U-47931E from 25 to 50 mg^13–15^. Despite being discussed in drug user forums since U-47700 was subjected to controls, both compounds have not been detected in forensic or clinical samples so far^16^, which may be due to the lack of information about suitable urine screening targets.

Another NSO, a fentanyl analogue, which has been associated with several fatal intoxications, is 2-methoxy-*N*-phenyl-*N*-[1-(2-phenylethyl)piperidin-4-yl] acetamide, commonly known as methoxyacetylfentanyl^17^. So far, fatalities in Sweden, Denmark, and the USA have been reported, together with numerous seizures throughout Europe^18^. According to drug user forums, the ingested dose of methoxyacetylfentanyl ranges from 0.5 to 5 mg depending on the route of administration^19,20^. However, no reports regarding potency are available yet^17,18^.

To understand and monitor intoxications with NSOs, knowledge of analytical targets is necessary for toxicological analysis. As urine is the preferred matrix for comprehensive screening, e.g. in abstinence monitoring programmes, doping analysis, workspace drug testing, and in the case of suspected intoxications, metabolites should be known.

Owing to the lack of such data, the aim of the present work was to elucidate the metabolic patterns of the synthetic non-fentanyl opioids U-51754 and U-47931E and of methoxyacetylfentanyl by using liquid chromatography high-resolution tandem mass spectrometry (LC-HR-MS/MS). Metabolic identification was performed *in vitro* using pooled human S9 fractions (phS9) and *in vivo* analysing rat urine after oral administration. Finally, both models were compared.

## Experimental

### Chemicals and reagents

Methoxyacetylfentanyl hydrochloride (purity ≥ 98%), U-51754 hydrochloride (purity 98%), and U-47931E were purchased from LGC Standards (Wesel, Germany). Isocitrate, isocitrate dehydrogenase, superoxide dismutase, 3′-phosphoadenosine-5′-phosphosulfate (PAPS), S-(5′-adenosyl)-L-methionine (SAM), dithiothreitol (DTT), reduced glutathione (GSH), acetyl carnitine, and acetyl coenzyme A (AcCoA) were all purchased from Sigma (Taufkirchen, Germany), NADP^+^ from Biomol (Hamburg, Germany), and acetonitrile (LC-MS grade), ammonium formate (analytical grade), formic acid (LC-MS grade), methanol (LC-MS grade), glucuronidase (EC No. 3.2.1.32)/arylsulfatase (EC No. 3.1.6.1) from Helix pomatia L, and all other chemicals and reagents (analytical grade) from VWR (Darmstadt, Germany). phS9 (20 mg protein/mL, from 30 individual donors), UGT reaction mix solution A (25 mM UDP-glucuronic acid), and UGT reaction mix solution B (250 mM Tris–HCl, 40 mM MgCl_2_, and 0.125 mg/mL alamethicin) were obtained from Corning (Amsterdam, The Netherlands). After delivery, the enzyme preparations were thawed at 37 °C, aliquoted, snap-frozen in liquid nitrogen, and stored at −80 °C until use.

### Incubation conditions for identification of phase I and II metabolites by LC-HR-MS/MS in phS9

The incubation conditions were in accordance to the experimental design developed by Richter *et al*.^21^. Incubations of phS9 (final protein concentration of 2 mg/mL) were performed after 10 min pre-incubation at 37 °C with 25 μg/mL alamethicin (UGT reaction mix solution B), 90 mM phosphate buffer (pH 7.4), 2.5 mM Mg^2+^, 2.5 mM isocitrate, 0.6 mM NADP^+^, 0.8 U/mL isocitrate dehydrogenase, 100 U/mL superoxide dismutase, 0.1 mM AcCoA, and 2.3 mM acetyl carnitine. Afterwards, to reach a final volume of incubation mixture of 150 µL, 2.5 mM UDP-glucuronic acid (UGT reaction mix solution A), 40 μM aqueous PAPS, 1.2 mM SAM, 1 mM DTT, 10 mM GSH, and 25 μM substrate in phosphate buffer were added. All given concentrations are final concentrations. By adding the substrate, the reaction was initiated and the mixture was incubated for 60 and 360 min respectively. After 60 min a 60 µL aliquot of the mixture was transferred into a reaction tube. Reactions were terminated by adding 20 µL of ice-cold acetonitrile. The remaining mixture was incubated for additional 5 h. Thereafter, the reactions were stopped by adding 30 µL of ice-cold acetonitrile. The solutions were cooled for 30 min at −18 °C and centrifuged for 2 min at 14,000 rpm. The supernatants were transferred into autosampler vials and 5 µL injected onto the Orbitrap-based LC-HR-MS/MS system as described below. To confirm the absence of interfering compounds and to identify non-metabolically originated compounds, additional blank incubations without substrate and control samples without phS9 were prepared.

### Rat urine samples

According to an established study design^22–24^, the investigations were performed using rat urine samples from male Wistar rats (Charles River, Sulzfeld, Germany) for toxicological diagnostic reasons according to German law. The experimental protocol was approved by an ethics committee (Landesamt für Verbraucherschutz, Saarbrücken, Germany). For metabolite identification, the compounds were administered in an aqueous suspension by gastric intubation of a single 0.6 mg/kg (U-47931E, U-51754) or 0.06 mg/kg (methoxyacetylfentanyl) body weight dose, calculated based on doses reported in trip reports and scaled by dose-by-factor approach from man to rat according to Sharma and McNeill^16^. The rats were housed in metabolism cages for 24 h, having water ad libitum. Urine was collected separately from faeces over a 24 h period using a grid plate. Blank urine samples were collected before drug administration to verify that the samples were free of interfering compounds. The samples were aliquoted and stored at −20 °C before analysis.

### Sample preparation for identification of phase I and II metabolites by LC-HR-MS/MS in rat urine

In accordance to published procedures^25^, 100 µL of urine was mixed with 500 µL of acetonitrile for precipitation. After shaking and centrifugation for 2 min at 14,000 rpm, the supernatant was transferred into new vials and gently evaporated to dryness under a stream of nitrogen. Afterwards, the extract was reconstituted in 50 µL of eluent A and eluent B (1:1, v/v) and transferred into autosampler vials.

According to previous studies^26^, additionally, urine samples were treated with β-glucuronidase/arylsulfatase for conjugate cleavage prior to the extraction procedure. A volume of 200 µL urine was incubated with 20 µL β-glucuronidase/arylsulfatase and 180 µL 100 mM aqueous ammoniumacetate buffer (pH 5.2) for 2 h at 55 °C. The extract was precipitated by adding 1 mL of acetonitrile and stored for 5 min at −20 °C. After shaking and centrifugation, the supernatant was gently evaporated and reconstituted with 100 µL of mobile phase A and B (1:1, v/v) described below.

Furthermore, a basic solid phase extraction (SPE) was performed^23^. SPE was carried out using Biotage HCX columns (Isolute Confirm HXC cartridges, 130 mg, 3 mL from Biotage, Uppsala, Sweden). They were conditioned with 1 mL of methanol and 1 mL of water. An aliquot of 2.5 mL urine was mixed with 2 mL of water and 50 µL of internal standard (0.01 mg/mL trimipramine-d_3_) and loaded onto the cartridges. Two washing steps with 1 mL water and 1 mL HCl (0.01 M) were performed. Maximum vacuum was applied for a short time. After adding 2 mL of methanol, the columns were dried for one min under maximum vacuum. Analyte elution was performed with 1 mL methanol/33% aqueous ammonia (98:2 v/v). The eluate was evaporated to dryness under a gentle stream of nitrogen at 70 °C and reconstituted with 50 µL of methanol. For conjugate cleavage, additional urine aliquots were treated with glucuronidase/arylsulfatase prior to the extraction procedure. An aliquot of 2.5 mL urine was adjusted to pH 5.2 with 1 M acetic acid. After incubation with 50 µL glucuronidase/arylsulfatase for 2 h at 55 °C following centrifugation, SPE was performed as described above. Five microlitres of the extracts were injected onto the LC-HR-MS/MS system with conditions described below.

### LC-HR-MS/MS instrumentation for identification of phase I and II metabolites

As described previously^27^, the extracts were analysed using a Thermo Fisher Scientific (TF, Dreieich, Germany) Dionex UltiMate 3000 RS pump consisting of a degaser, a quaternary pump, and an UltiMate autosampler, coupled to a TF Q-Exactive Plus system equipped with a heated electrospray ionization (HESI)-II source.

The LC conditions were as follows: Gradient elution was run on a TF Accucore PhenylHexyl column (100 mm × 2.1 mm, 2.6 µm) with column oven temperature of 60 °C. The mobile phase consisted of 2 mM aqueous ammonium formate containing 0.1% (v/v) formic acid and 1% (v/v) acetonitrile (pH 3, eluent A), and 2 mM ammonium formate solution with acetonitrile/methanol (50:50, v/v) containing 0.1% (v/v) formic acid and 1% (v/v) water (eluent B). The gradient and flow rate were programmed as follows: 0–1 min hold 99% A, 1–10 min 99% A to 1% A, both with flow rate 0.5 mL/min, 10–11.5 min hold, 11.5–13.5 min hold 99% A, both with flow rate 0.8 mL/min.

The HESI-II source conditions were as follows: sheath gas, 60 arbitrary units (AU); auxiliary gas, 10 AU; spray voltage, 3.00 kV; heater temperature, 320 °C; ion transfer capillary temperature, 320 °C; and S-lense RF level, 60.0.

The mass spectrometry was operated in positive ionisation mode using full scan (FS) and subsequent data dependent acquisition (DDA) mode with inclusion list containing the *m/z* values of expected metabolites. Mass calibration was done prior to analysis according to the manufacture’s recommendations using external mass calibrators. The settings for FS data were as follows: resolution, 35,000; microscans, 1; automatic gain control (AGC) target, 1e6; maximum injection time (IT), 120 ms; scan range for U-compounds, *m/z* 100–600 and for methoxyacetylfentanyl, *m/z* 180–600. The settings for the DDA mode were as follows: option “do not pick others”, enabled; dynamic exclusion, off; resolution, 17,500; microscans, 1; loop count, 5; AGC target, 2e5; maximum IT, 250 ms; isolation window, 1.0 *m/z*, high collision dissociation (HCD) with stepped normalised collision energy (NCE), 17.5, 35, and 52.5%; spectrum data type, profile; underfill ratio, 0.5%. For data handling Xcalibur Qual Browser software version 3.0.63 was used. The MS settings as well as the LC conditions were the same for analysing all samples.

## Results

### ESI^+^ HR-MS/MS of the investigated compounds and tentative identification of their phase I metabolites based on MS/MS fragmentation

**Figure 1:**
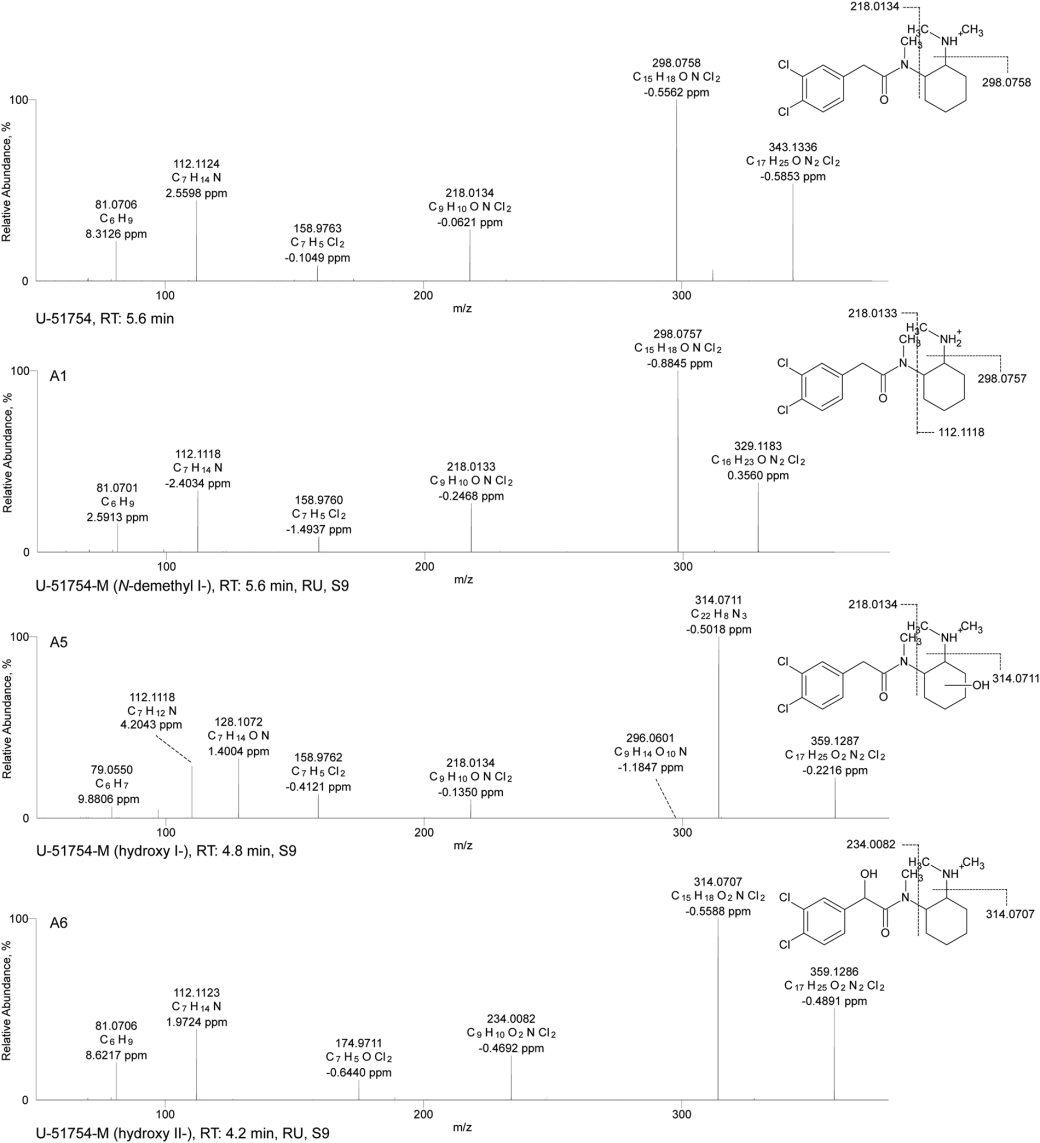
HR-MS/MS spectra of U-51754 and selected phase I metabolites. The spectra with proposed structures, retention times (RT), detected in rat urine (RU) and/or pooled human S9 fraction incubations (S9), and predominant fragmentation patterns of U-51754 and metabolites are arranged according to their presentation in the text.

**Figure 2:**
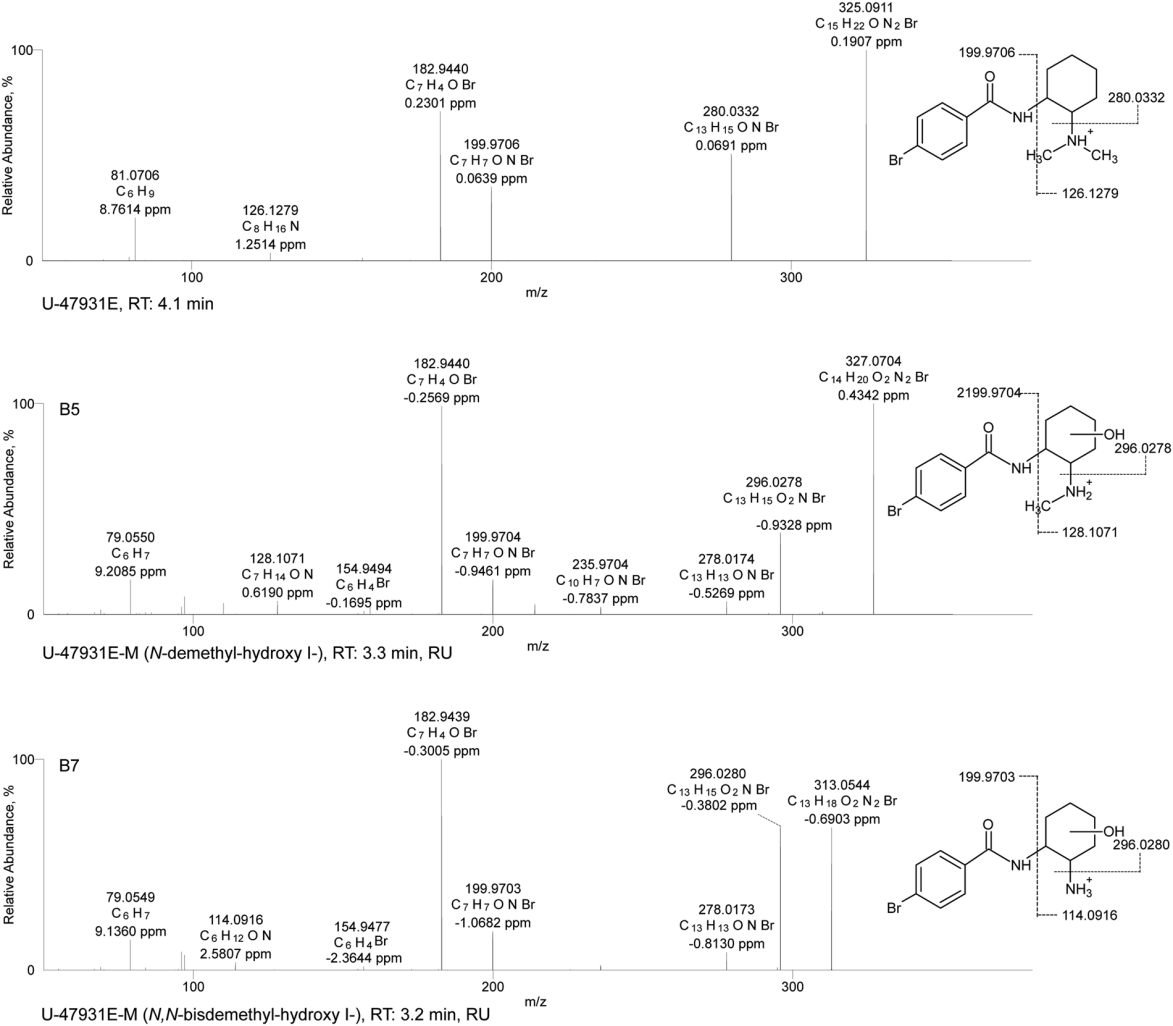
HR-MS/MS spectra of U-47931E and selected phase I metabolites. The spectra with proposed structures, retention times (RT), detected in rat urine (RU) and/or pooled human S9 fraction incubations (S9), and predominant fragmentation patterns of U-47931E and metabolites are arranged according to their presentation in the text.

**Figure 3:**
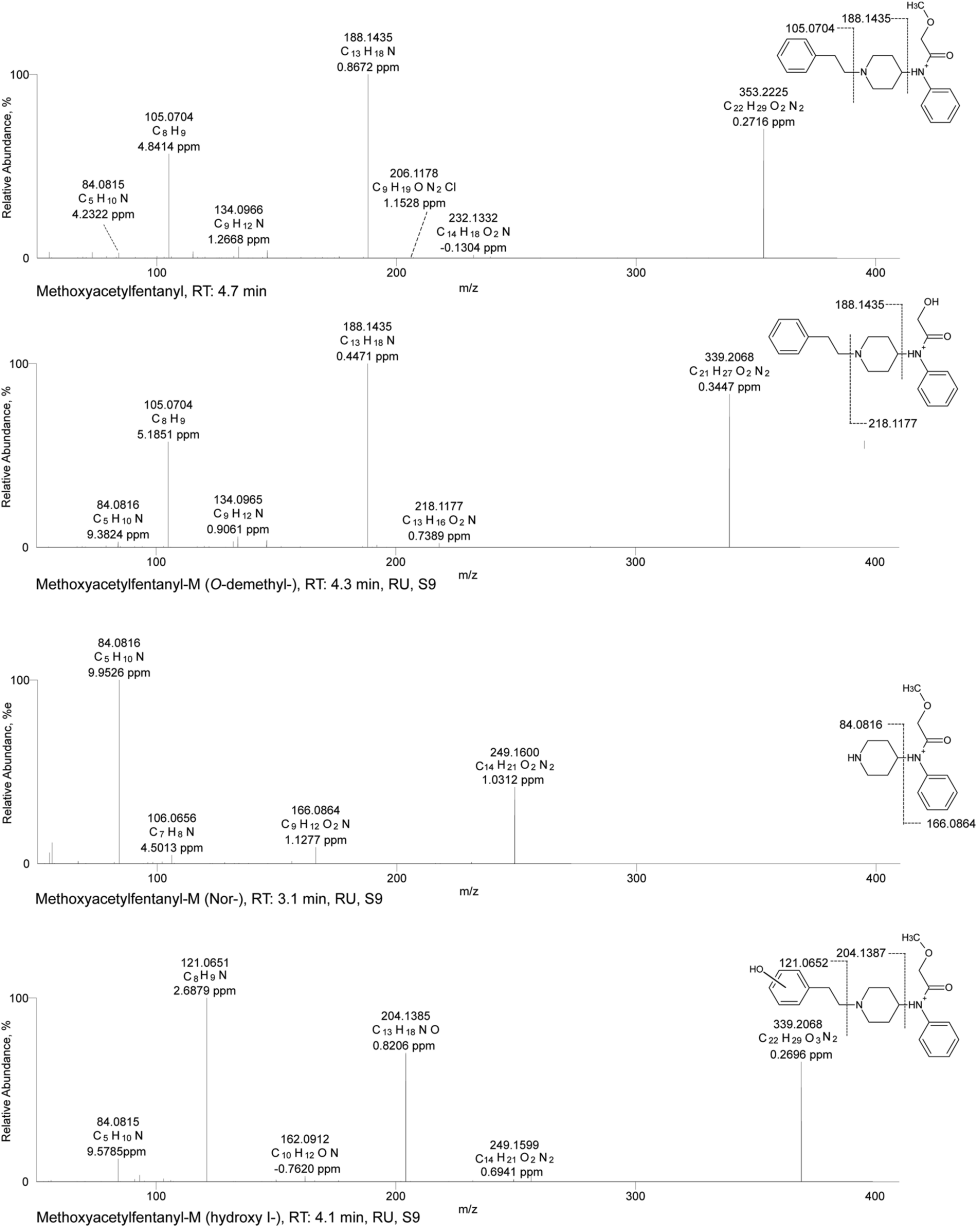
HR-MS/MS spectra of methoxyacetylfentanyl and selected phase I metabolites. The spectra with proposed structures, retention times (RT), detected in rat urine (RU) and/or pooled human S9 fraction incubations (S9), and predominant fragmentation patterns of methoxyacetylfentanyl and metabolites are arranged according to their presentation in the text.

An overview of all tentatively identified phase I and II metabolites is given in Tables 1–3, including elemental compositions, exact masses, accurate masses, characteristic fragment ions, mass errors, and retention times. Selected ESI^+^ MS^2^ spectra of the most abundant metabolites are shown in Figs 1–3. Selected mass spectral data for additional metabolites are shown in the Electronic Supplementary Material (EMS). Owing to the high number of tentatively identified metabolites, not all are discussed in detail in the following. The numbering of given examples is in accordance with the respective tables.

**Table 1:** Identification (ID), U-51754 and its metabolites as well as elemental composition, exact mass, accurate mass, characteristic product ions, mass errors, and retention time (RT) of the compounds detected in pS9 incubations and in rat urine after oral administration.

ID	Analyte	Elemental composition	Monoisotopic exact masses	Monoisotopic accurate masses	Error (ppm)	Accurate fragment masses (*m/z*)								
						Product 1	Error (ppm)	Product 2	Error (ppm)	Product 3	Error (ppm)	Product 4	Error (ppm)	RT (min)
	**U-51754**	C_17_H_25_ON_2_Cl_2_	343.1338	343.1336	−0.585	298.0758	−0.556	218.0134	−0.062	158.9763	−0.105	112.1124	2.559	5.62
A1	*N*-demethyl I-	C_16_H_23_ON_2_Cl_2_	329.1181	329.1183	0.356	298.0757	−0.885	218.0133	−0.247	158.9760	−1.494	112.1118	−2.403	5.60
A2	*N*-demethyl II-	C_16_H_23_ON_2_Cl_2_	329.1181	329.1177	−1.485	284.0609	1.905	203.9982	2.037	158.9767	3.892	98.0969	4.349	5.47
A3	*N*,*N*-bisdemethyl-	C_15_H_21_ON_2_Cl_2_	315.1025	315.1020	−1.580	284.0602	−0.401	203.9977	−0.124	158.9765	1.649	98.0968	3.684	5.42
A4	*N*,*N*,*N*-tridemethyl-	C_14_H_20_ON_2_Cl_2_	301.0868	301.0872	0.964	284.0604	0.268	203.9980	1.076	158.9763	0.130	98.0970	5.803	5.22
A5	Hydroxy I-	C_17_H_25_O_2_N_2_Cl_2_	359.1287	359.1287	−0.221	314.0711	0.538	218.0134	−0.135	158.9762	−0.412	110.0969	4.204	4.75
A6	Hydroxy II-	C_17_H_25_O_2_N_2_Cl_2_	359.1287	359.1286	−0.489	314.0707	−0.559	234.0082	−0.469	174.9711	−0.644	112.1123	1.972	4.15
A7	Dihydroxy I-	C_17_H_25_O_3_N_2_Cl_2_	375.1236	375.1214	−6.067	330.0651	−2.170	234.0079	−1.840	174.9709	−1.863	110.0968	3.145	3.32
A8	Dihydroxy II-	C_17_H_25_O_3_N_2_Cl_2_	375.1236	375.1536	3.050	330.0927	1.001	250.0273	2.964	190.9890	3.223	112.1124	2.924	5.21
A9	*N*-oxide-	C_17_H_25_O_2_N_2_Cl_2_	359.1287	359.1292	1.130	298.0947	−1.128	218.0132	−0.855	158.9766	2.265	112.1124	2.860	5.52
A10	*N*-oxide-hydroxy-	C_17_H_25_O_3_N_2_Cl_2_	375.1236	375.1236	−0.316	314.0710	0.416	218.0134	−0.108	158.9762	−0.338	110.0969	4.204	4.90
A11	*N*-demethyl-hydroxy I-	C_16_H_23_O_2_N_2_Cl_2_	345.1131	345.1130	−0.424	314.0707	−0.083	218.0133	−0.103	158.9761	−1.083	110.0966	1.236	4.86
A12	*N*-demethyl-hydroxy II-	C_16_H_23_O_2_N_2_Cl_2_	345.1131	345.1128	−0.963	314.0709	−0.041	234.0083	−0.000	174.9711	−0.819	112.1123	2.158	4.49
A13	*N*,*N*-bisdemethyl-hydroxy I-	C_15_H_21_O_2_N_2_Cl_2_	331.0974	331.0977	0.7805	314.0711	0.449	203.9978	0.145	158.9763	−0.074	79.055	9.865	4.77
A14	*N*,*N*-bisdemethyl-hydroxy II-	C_15_H_21_O_2_N_2_Cl_2_	331.0974	331.0976	0.410	314.0708	−0.413	219.9925	−0.690	174.9711	−0.434	112.1123	2.191	4.48
A15	hydroxy-glucuronide	C_23_H_33_O_8_N_2_Cl_2_	535.1608	535.1611	0.471	359.1273	−1.253	314.0707	0.736	234.0082	−0.546	112.1123	1.996	4.58
A16	dihydroxy-glucuronide	C_23_H_33_O_9_N_2_Cl_2_	551.1557	551.1549	−1.536	375.1234	−0.705	330.0695	0.157	250.9661	−0.080	112.1123	2.087	3.76
A17	*N*-demethyl-hydroxy-glucuronide	C_22_H_31_O_8_N_2_Cl_2_	521.1451	521.1451	−0.101	345.1129	−0.534	314.0706	0.631	234.0082	−0.673	112.1123	2.089	3.59
A18	*N*-demethyl-hydroxy-sulfate	C_16_H_23_O_5_N_2_Cl_2_S	425.0699	425.0685	−3.257	394.0266	−2.983	345.1121	−2.736	314.0701	−2.398	112.1121	−0.114	4,23
A19	*N*-demethyl-dihydroxy-glucuronide	C_22_H_30_O_9_N_2_Cl_2_	537.1401	537.1386	−2.828	361.1070	−3.280	330.0649	−2.773	250.0025	−3.260	112.1121	−0.054	3.82
A20	*N*,*N*-bisdemethyl-hydroxy-glucuronide	C_21_H_28_O_8_N_2_Cl_2_	507.1295	507.1260	4.656	331.0975	0.028	300.0554	0.548	219.9925	−0.938	112.1124	2.466	3.59
A21	*N*,*N*-bisdemethyl-hydroxy-sulfate	C_15_H_20_O_5_N_2_Cl_2_S	411.0542	411.0529	−5.071	331.0962	−4.097	300.0542	−2.954	219.9921	−1.624	112.1122	0.746	4.15

Elemental composition and all given masses are protonated forms.

**Table 2:** Identification (ID), U-47931E and its metabolites as well as elemental composition, exact mass, accurate mass, characteristic product ions, mass errors, and retention time (RT) of the compounds detected in pS9 incubations and in rat urine after oral administration.

ID	Analyte	Elemental composition	Monoisotopic exact masses	Monoisotopic accurate masses	Error (ppm)	Accurate fragment masses (*m/z*)								
						Product 1	Error (ppm)	Product 2	Error (ppm)	Product 3	Error (ppm)	Product 4	Error (ppm)	RT (min)
	U-47931E	C_15_H_22_ON_2_Br	325.0909	325.0911	0.191	280.0332	0.069	199.9706	0.064	182.9440	0.230	126.1279	1.251	4.10
B1	*N*-demethyl-	C_14_H_20_ON_2_Br	311.0753	311.0752	−0.392	280.0331	−0.095	199.9705	−0.061	182.9441	0.272	112.1124	3.325	3.98
B2	*N*,*N*-bisdemethyl-	C_13_H_18_ON_2_Br	297.0596	297.0594	−0.919	280.0327	−1.551	199.9702	0.103	182.9437	−1.391	98.0969	4.346	3.89
B3	Hydroxy I-	C_15_H_22_O_2_N_2_Br	341.0859	341.0858	−0.313	296.0278	−0.973	278.0174	−0.434	182.9439	−0.433	142.1226	−0.373	3.45
B4	Hydroxy II-	C_15_H_22_O_2_N_2_Br	341.0859	341.0860	0.238	296.0280	−0.075	215.9654	−0.133	198.9388	−0.610	126.1226	0.737	4.38
B5	*N*-demethyl-hydroxy I-	C_14_H_20_O_2_N_2_Br	327.0702	327.0704	0.434	296.0278	−0.933	278.0174	−0.027	182.9440	−0.4257	128.1071	0.619	3.30
B6	*N*-demethyl-hydroxy II-	C_14_H_20_O_2_N_2_Br	327.0702	327.0706	1.061	296.0284	1.101	215.9655	0.092	198.9389	−0.024	112.1124	2.969	4.19
B7	*N*,*N*-bisdemethyl-hydroxy I-	C_13_H_18_O_2_N_2_Br	313.0546	313.0544	−0.690	296.0280	−0.380	278.0173	−0.813	182.9439	−0.301	114.0916	2.581	3.15
B8	*N*,*N*-bisdemethyl-hydroxy II-	C_13_H_18_O_2_N_2_Br	313.0546	313.0500	1.610	296.0283	0.718	215.9655	0.718	198.9388	−0.809	98.0970	5.462	4.14
B9	*N*-demethyl-dihydroxy-	C_14_H_20_O_3_N_2_CBr	343.0651	343.0646	−1.738	312.0227	−0.899	231.9600	−1.527	214.9336	−1.070	112.1124	2.745	4.34
B10	Hydroxy-glucuronide	C_21_H_30_O_8_N_2_Br	517.1179	517.1186	1.139	341.0863	1.022	296.0282	0.370	215.9655	0.049	126.1278	0.737	3.77
B11	Dihydroxy-glucuronide	C_21_H_30_O_9_N_2_Br	533.1129	533.1129	0.016	312.0227	−0.905	231.9604	0.216	214.9337	−0.803	112.1124	2.839	4.35
B12	*N*-demethyl-hydroxy-glucuronide	C_20_H_28_O_8_N_2_Br	503.1023	503.1029	1.103	327.0704	0.431	296.0282	0.498	198.9388	−0.683	112.1124	2.563	3.65
B13	*N*,*N*-bisdemethyl-hydroxy-glucuronide	C_19_H_26_O_8_N_2_Br	489.0866	489.0866	−0.165	313.0544	−0.642	296.0283	0.718	215.9654	−0.199	198.9388	−0.643	3.58

Elemental composition and all given masses are protonated forms.

**Table 3:** Identification (ID), methoxyacetylfentanyl and its metabolites as well as elemental composition, exact mass, accurate mass, characteristic product ions, mass errors, and retention time (RT) of the compounds detected in pS9 incubations and in rat urine after oral administration.

ID	Analyte	Elemental composition	Monoisotopic exact masses	Monoisotopic accurate masses	Error (ppm)	Accurate fragment masses (*m/z*)								
						Product 1	Error (ppm)	Product 2	Error (ppm)	Product 3	Error (ppm)	Product 4	Error (ppm)	RT (min)
	Methoxyacetylfentanyl	C_22_H_29_O_2_N_2_	353.2223	353.2225	0.272	188.1435	0.867	134.0966	1.267	105.0704	4.841	84.0811	4.232	4.69
C1	*N*-dealkyl- (Nor)	C_14_H_21_O_2_N_2_	249.1597	249.1600	1.031	166.0864	1.128	106.0656	4.501	84.0816	9.953			3.07
C2	*N*-dealkyl-*O*-demethyl-	C_13_H_19_O_2_N_2_	235.1441	235.1442	−0.600	152.0707	0.878	94.0657	5.665	81.0812	9.881			1.84
C3	Amide hydrolyzed-	C_19_H_25_N_2_	281.2012	281.2014	0.679	188.1434	0.187	134.0965	0.823	105.0703	3.931	84.0811	3.675	4.98
C4	Amide hydrolyzed hydroxy I-	C_19_H_25_ON_2_	297.1961	297.1963	0.426	205.1337	0.554	188.1438	2.238	134.096	1.619	105.0706	6.817	3.06
C5	Amide hydrolyzed hydroxy II-	C_19_H_25_ON_2_	297.1961	297.1962	0.211	204.1385	1.053	121.0651	2.394	84.0815	8.749			4.41
C6	*O*-demethyl-	C_21_H_25_O_2_N_2_	339.2067	339.2068	0.345	218.1177	0.739	188.1435	−0.447	134.0965	0.906	105.0704	5.185	4.31
C7	*O*-demethyl-hydroxy I-	C_21_H_25_O_3_N_2_	355.2016	355.2020	1.033	235.1439	−0.705	204.1385	1.022	121.0651	2.509	84.0815	8.876	3.65
C8	*O*-demethyl-hydroxy II-	C_21_H_25_O_3_N_2_	355.2016	355.2016	−0.056	337.1910	−0.133	204.1384	0.446	186.1272	−2.796	91.0548	5.897	3.94
C9	*O*-demethyl-hydroxy III-	C_21_H_25_O_3_N_2_	355.2016	355.2018	1.046	188.1434	0.183	134.0965	0.874	105.0703	3.831	84.0815	8.475	4.01
C10	*O*-demethyl-hydroxy-methoxy-	C_22_H_29_O_4_N_2_	385.2121	385.2124	0.823	234.1488	−0.389	192.1019	−0.105	151.0756	1.330	119.0494	2.289	3.87
C11	Hydroxy I-	C_22_H_29_O_3_N_2_	369.2172	369.2176	0.795	249.1598	0.089	204.1385	0.968	121.0651	2.270	84.0815	8.648	4.09
C12	Hydroxy II-	C_22_H_29_O_3_N_2_	369.2172	369.2175	0.704	351.2068	0.322	204.1383	0.073	186.1272	−3.071	105.0703	4.436	4.38
C13	*N*-oxide I-	C_22_H_29_O_3_N_2_	369.2172	369.2180	2.020	261.1598	0.136	189.1386	0.085	146.0965	0.170	105.0703	4.218	5.10
C14	*N*-oxide II-	C_22_H_29_O_3_N_2_	369.2172	369.2178	1.382	261.1599	0.596	186.1279	0.786	158.0965	0.309	105.0703	4.366	5.22
C15	Hydroxy-methoxy-	C_23_H_31_O_4_N_2_	399.2278	575.2580	0.528	234.1490	0.441	151.0755	1.054	119.0494	2.016	84.0815	8.082	4.19
C16	Amide hydrolysed hydroxy-glucuronide	C_25_H_33_O_7_N_2_	473.2282	473.2283	0.214	297.1963	0.540	188.1436	1.229	105.0705	4.800			3.06
C17	*O*-demethyl-glucuronide	C_27_H_35_O_8_N_2_	515.2387	515.2393	0.952	339.2069	0.444	218.1177	0.436	188.1435	0.759	105.0703	4.500	4.03
C18	*O*-demethyl-hydroxy-glucuronide	C_27_H_35_O_9_N_2_	531.2387	531.2340	0.496	355.2022	1.635	204.1386	1.716	121.0652	3.012	84.0816	9.696	3.47
C19	*O*-demethyl-hydroxy-methoxy-glucuronide	C_29_H_35_O_10_N_2_	561.2442	561.2447	0.696	385.2124	0.681	234.1488	−0.331	151.0755	0.995	119.0493	0.979	3.31
C20	Hydroxy-glucuronide	C_28_H_35_O_9_N_2_	545.2493	545.2501	1.314	369.2177	1.221	249.1600	1.123	204.1386	1.523	121.0652	3.047	3.65
C21	Hydroxy-methoxy-glucuronide	C_29_H_39_O_10_N_2_	575.2599	575.2599	−0.089	399.2281	0.684	234.1491	0.881	192.1019	0.111	151.0755	0.757	3.73

Elemental composition and all given masses are protonated forms.

As already described for U-47700 in previous metabolism studies^28^, the HR-MS data indicated that U-51754 and U-47931E could be broken down into two parts at the amide nitrogen usually forming fragment ions that belong to the dichlorophenyl-*N*-methylacetamide part or the cyclohexyl part. The fragment ions of the unmodified or modified dichlorophenyl part or the cyclohexyl part were used (according to Figs 1 and 2 and Tables 1 and 2) for the spectra interpretation and identification of the expected metabolites based on the accurate precursor mass (PM) and calculated molecular formulas. Based on the procedure described, 14 phase I metabolites of U-51754 could be identified. In phS9 incubates, 10 metabolites were detected (A1–6, A9-11, A13). In these, *N*-demethyl-U-51754 formed the most abundant peak. In rat urine, seven metabolites were observed (A1, A6-8, A11-12, A14), with *N*-demethyl-hydroxy-U-51754 being the main metabolite. In urine samples, seven additional phase II metabolites were found (A15-21), five conjugated with glucuronic acid and two with sulphuric acid. Nine phase I metabolites of U-47931E could be identified. In this connection, *N*-demethyl-U-47931E was the most abundant peak in phS9 incubates and rat urine samples. *In vitro*, three metabolites were found (B1-3), and *in vitro* all nine (B1-9) metabolites were detected. In the urine samples, four additional glucuronides were identified (B10-13).

The HR-MS data of methoxyacetylfentanyl indicated that the molecule could be cleaved between the piperidine ring and the *N*-phenylacetamide moiety leading to the most abundant fragment ion of the phenylethylpiperidine. The phenethyl chain represented another characteristic fragment ion. The fragment ions of the unmodified or modified phenylethylpiperidine, phenethyl chain or methoxy-*N*-phenylacetamide were used (according to Fig. 3 and Table 3) for the spectra interpretation of methoxyacetylfentanyl metabolites. In total, 15 phase I metabolites could be tentatively identified for methoxyacetylfentanyl. In phS9 incubates, 14 metabolites were detected (C1-9, C11-15) with *O*-demethyl-methoxyacetylfentanyl forming the most abundant peak. In rat urine, 11 metabolites were observed (C1-2, C4, C6-8, C10-12, C14-15), with hydroxy-methoxyacetylfentanyl being the main metabolite. In urine samples, six additional glucuronides were found (C16-21). One of them could be identified in phS9 as well.

### ESI^+^ HR-MS/MS fragmentation of U-51754

U-51754 (PM at *m/z* 343.1336) was characterised by the most abundant fragment ion at *m/z* 298.0758 (C_15_H_18_ONCl_2_), indicating loss of the dimethylamine moiety. The 3,4-dichlorophenyl-*N*-methylacetamide (*m/z* at 218.0134, C_9_H_10_ONCl_2_) and the *N*-methylcyclohexanamine (*m/z* at 112.1124, C_7_H_14_N) were other characteristic fragment ions. Low-abundant fragment ions at *m/z* 158.9763 and 81.0707 were related to the 3,4-dichloro-1-methylbenzene and the cyclohexyl ring respectively.

### N-demethyl-U-51754

According to Fig. 1, the fragmentation pattern of the most abundant metabolite, *N*-demethyl-U-51754 (**A1**, PM at *m/z* 329.1183), showed, as expected, an initial loss of methanamine forming the most abundant fragment ion at *m/z* 298.0757 followed by the cleavage at the amide nitrogen producing the fragment ion at *m/z* 218.0133, which belongs to the dichlorophenyl-*N*-methylacetamide part. Furthermore, the metabolite was identified by a fragmentation pattern similar to that of the parent substance, indicating loss of methyl at the amine moiety. *N*-demethyl-U-51754 formed two isomers in phS9 incubations, which could be separated chromatographically. Thus, the position of *N*-demethylation must be different. The spectrum of **A2** (EMS) represented fragment shifts due to the loss of a methyl group (−14.0156 u) at the fragments related to the dichlorophenyl-*N*-methylacetamide and the cyclohexyl-dichlorophenyl-*N*-methylacetamide.

### Hydroxy-U-51754

The hydroxy metabolite **A5** (PM at *m/z* 359.1287) was identified by the parent ion as well as the fragment ions at *m/z* 314.0711, representing the loss of dimethylamine at the cyclohexyl ring. The fragment ions at *m/z* 218.0134 and 158.9762 corresponded to an unaltered phenyl-*N*-methylacetamide. However, the presence of the fragment ions at *m/z* 79.0550 and 110.0969 indicated the loss of water after hydroxylation at the cyclohexyl ring. Taking these findings together, this spectrum might be the result of a monohydroxylation at the cyclohexyl ring. Regarding this PM and those of other hydroxylated metabolites, multiple peaks occurred in the chromatogram, which implicated sites of hydroxylation at the cyclohexyl ring. However, it was not possible to determine the exact position of hydroxylation and its relationship to different retention times. Regarding monohydroxylation, several metabolites could be identified. The first peak with the PM of *m/z* 359.1287 was related to a hydroxylation that occurred at the benzyl ring or the methylene linker (**A6**). **A6** was identified by the characteristic fragment ions at *m/z* 81.0706 and 112.1123, indicating an unaltered cyclohexyl ring and thus excluding this as the site of hydroxylation. Additionally, no loss of water was observed after cleavage of the hydroxy group. However, the ions at *m/z* 174.9711 and 234.0082 with a mass shift of +15.9946 u (O) indicated a hydroxylation at the dichloro-methylbenzene moiety. Thus, this spectrum might be the result of monohydroxylation at either the benzyl ring or the methylene linker. The exact position of the hydroxy group could not be elucidated by the methods applied.

### ESI^+^ HR-MS/MS fragmentation of U-47931E

U-47931E (PM of *m/z* 325.0911) showed a concise fragmentation pattern. The 4-bromobenzaldehyde moiety represented the most abundant fragment ion at *m/z* 182.9940 (C_7_H_5_OBr). Loss of dimethylamine and the 4-bromobenzamide led to other characteristic fragment ions at *m/z* 280.0322 (C_13_H_18_ONBr) and 199.9706 (C_7_H_7_ONBr). Low-abundant fragment ions at *m/z* 81.0705 (C_6_H_9_) and 126.1279 (C_8_H_16_N) were related to the cyclohexyl ring and the dimethyl cyclohexanamine respectively.

### *N*-demethyl-hydroxy-U-47931E and *N,N*-bisdemethyl-hydroxy-U-47931E

According to Fig. 2, the *N*-demethylated hydroxylated metabolite (**B5**, PM at *m/z* 327.0704) was identified by the fragment ion at *m/z* 296.0278, showing a loss of the methylamine and the characteristic mass shift of a hydroxy group (+15.9946 u, O). Furthermore, the concise fragment ions such as 182.9440 and 199.9704 were also present in the parent spectrum, indicating an unaltered bromobenzamide part. The fragment ions at *m/z* 79.0550 and 278.0174 resulted from loss of water at the cyclohexyl ring. Taking these findings together, this spectrum seemed to be related to an *N*-demethylated metabolite with further monohydroxylation at the cyclohexyl ring. The same characteristics were observed for the *N*,*N*-bisdemethylated hydroxylated metabolite (**B7**, PM at *m/z* 313.0544), which showed a similar fragmentation pattern to **B5** except for the precursor ion. Thus, hydroxylation must occur at the cyclohexyl ring, too. Regarding hydroxylated metabolites, multiple peaks occurred in the chromatogram, implicating multiple sites of hydroxylation at the cyclohexyl ring. Concerning the structure of the metabolite, it should be mentioned that the exact position of the hydroxy group in the cyclohexyl ring could not be deduced from the fragmentation pattern and with the analytical methods used here.

Additionally, after SPE, minor amounts of all hydroxylated metabolites could be detected, with hydroxylation occurring at the benzyl instead of the cyclohexyl ring (**B4**, **B6**, **B8**, EMS). All fragmentation patterns showed the characteristic mass shift due to the presence of a hydroxy group at fragments related to the bromobenzyl ring, indicating monohydroxylation at the aromatic system.

### ESI^+^ HR-MS/MS fragmentation of methoxyacetylfentanyl

The HR-MS data of methoxyacetylfentanyl (PM of *m/z* 353.2225) formed the most abundant fragment ion at *m/z* 188.1435 (C_13_H_18_N). The phenethyl chain represented another characteristic fragment at *m/z* 105.0704 (C_8_H_9_). More fragment ions in low abundance were observed in the spectrum The fragment ion at *m/z* 84.0811 represented the intact piperidine ring, whereas degradation of the piperidine ring led to the minor fragment ions at *m/z* 134.0966 (C_9_H_12_N), 146.0958 (C_10_H_12_N), 206.1163 (C_12_H_16_O_2_N), and 232.1332 (C_14_H_18_O_2_N). Amide cleavage resulted in the fragment ion at *m/z* 166.0857 (2-methoxy *N*-phenylacetamide, C_9_H_12_O_2_N). Another low-abundant characteristic fragment ion was derived from the 4-anilio-*N*-phenethyl-piperidine part (4-ANPP) of the molecule (281.2014, C_19_H_25_N_2_) and could not be observed in all metabolite spectra. In general, methoxyacetylfentanyl showed a fragmentation pattern according to previous published metabolism studies of methoxyacetylfentanyl and other 4-anilinopiperidine-type fentanyl analogues^18,29,30^.

### *O*-demethyl-methoxyacetylfentanyl

The main metabolic pathway seemed to be *O*-demethylation (**C6**, PM of *m/z* 339.2068). According to Fig. 3, the MS^2^ spectrum showed the same characteristic fragment ions as the parent compound except for the mass of the parent compound showing a loss of a methyl group (−14.0156 u). As described above, the fragments of the phenylethylpiperidine, phenethyl chain or methoxy-*N*-phenylacetamide were used for identification. The fragment ion of the phenylethylpiperidine at *m/z* 188.1435 represented the unmodified part of the molecule. On the other hand, the methoxy-*N*-phenylacetamide showed loss of methyl (−14.0156 u), producing the fragment ion at *m/z* 218.1177 instead of 232.1332.

### Nor-methoxyacetylfentanyl

Compared with the parent compound, the nor-methoxyacetylfentanyl metabolite (*N*-dealkylated derivative, **C1**, PM at *m/z* 249.1600) exhibited only few fragment ions. However, this metabolite could be identified by characteristic fragment ions for the 2-methoxy *N*-phenylacetamide part of the molecule. Identification was carried out by the concise fragment ion at *m/z* 84.0816, related to the piperidine ring, as well as the fragment ion at *m/z* 166.0864, indicating an unchanged 2-methoxy *N*-phenylacetamide. Furthermore, no fragment ions belonging to the phenylethylpiperidine part of the molecule could be detected, indicating a cleavage of the molecule between the phenethylchain and the piperidine nitrogen.

### Hydroxy-methoxyacetylfentanyl

Regarding hydroxylation, several metabolites could be identified. Two monohydroxylated metabolites were found (**C11**, **C12**, PM at *m/z* 369.2179). **C11** showed the characteristic fragment ions of the phenylethylpiperidine part with a mass shift of oxygen (+15.9946 u, O) forming the fragment ions at *m/z* 121.0652 and 204.1387, indicating a hydroxylation at the phenethyl chain. Conversely, the hydroxy metabolite **C12** (EMS) showed initial loss of water forming the fragment ion at *m/z* 351.2054. The fragment ion at *m/z* 204.1387 represented hydroxylation at the phenylethylpiperidine part. However, the absence of the fragment ion at *m/z* 121.0652 and the presence of a mass shift of oxygen at the fragment at *m/z* 84.0815 resulted in the fragment at *m/z* 100.0763 and indicated a modified piperidine ring. Thus, hydroxylation might have occurred at the piperidine ring. Two other metabolites with the same *m/z* ratio were detected at 5.25 min (**C13**, **C14**, EMS) and could not be identified as hydroxy metabolites. Both spectra showed similar fragment ions to the parent spectrum, representing cleavage of oxygen without loss of water. These facts exclude the piperidine ring as the site of hydroxylation. The aromatic systems are also unlikely to undergo loss of hydroxy groups. Furthermore, the fragment ions formed are in accordance with those already described by Steuer *et al*. for *N*-oxide metabolites^31^. Those metabolites eluted after the parent compound, which has been frequently described for *N*-oxides^1,29,32^. Thus, *N*-oxidation at the nitrogen of the piperidine ring is the likeliest metabolic step. Owing to the molecular structure, two diastereomers could be formed with probably minimally different chromatographic properties, resulting in two detectable peaks.

### ESI^+^ HR-MS/MS for identification of the phase II metabolites based on MS/MS fragmentation

Most fragments in the spectra of the conjugates were also present in those of the corresponding phase I metabolites and are not discussed here. As shown in Tables 1–3, all glucuronides eliminated dehydrated glucuronic acid (−176.0321 u) and all sulphates dehydrated sulphuric acid (−79.9568 u). In total, seven corresponding phase II metabolites of U-51754 could be identified and four for U-47931E. Despite conjugate cleavage prior to extraction for some phase II metabolites (**A19**, **B11**, EMS), only trace amounts of the aglyca have been detected. However, the spectra of these metabolites showed characteristic fragment ions, so metabolite identification was possible. The aglycon of the hydroxy-glucuronide of U-47931E (**B10**, EMS) could not be identified in the urinary samples, possibly due to low stability. Nevertheless, reliable identification by characteristic fragment ions was possible for this metabolite. As described above, more than one isomer with similar MS^2^ spectra could be detected for all *N*-demethylated and hydroxylated metabolites of the U-substances. Caused by multiple sites of hydroxylation, more isomers could be formed after conjugation with glucuronic acid or sulphuric acid.

In total, six phase II metabolites of methoxyacetylfentanyl could be identified; all formed through conjugation of their corresponding phase I metabolite with glucuronic acid. For some phase II metabolites, fragment ions still containing the corresponding conjugate helped to evaluate the position of the conjugate. The fragment ion at *m/z* 380.1704 represented the hydroxy-4-ANPP conjugated with glucuronic acid after loss of the hydroxyacetaldehyde and could be found in the spectrum of the hydroxy-glucuronide (**C20**, EMS) and the *O*-demethyl-hydroxy-glucuronide (**C18**, EMS). The fragmentation ion at *m/z* 410.1818 represented the metabolised 4-ANPP part conjugated with glucuronic acid and could be found in the MS^2^ spectrum of the *O*-demethylated hydroxy-methoxy-glucuronide (**C19**, EMS). Sulphates of methoxyacetylfentanyl could not be detected, probably as a consequence of the generally reduced formation of sulphate conjugates in rats^30^. In line with our results, sulphates were only sparsely detected in previous studies as well^18^.

### Proposed metabolic pathways

**Figure 4:**
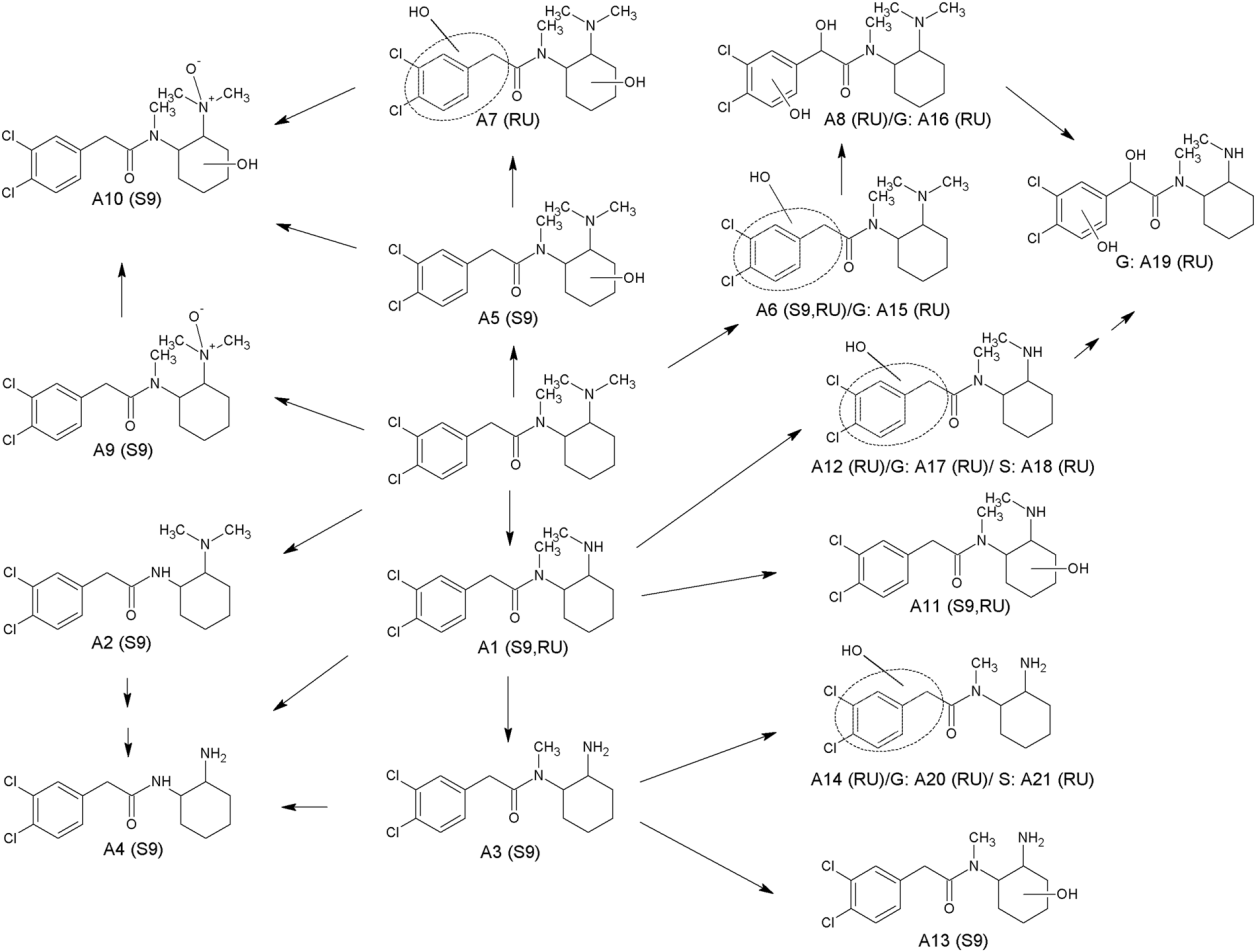
Metabolic pathways of U-51754 studied in rat urine (RU) and pooled human S9 fraction incubations (S9). Phase II metabolites: glucuronides (G), and sulfates (S). Numbering according to Table 1.

The proposed metabolic pathways of U-51754 are shown in Fig. 4. The predominant pathway was single or multiple *N*-demethylation of the amine moiety (**A1**, **A2**, **A3**, **A4**) and hydroxylation of the cyclohexyl ring (**A5**). Hydroxylation to the respective *N*-oxide (**A9**) was also observed, as well as further hydroxylation of this metabolite at the hexyl ring (**A10**). After *N*-demethylation, the metabolites underwent further oxidation to the respective hydroxy derivates (**A11**, **A13**). Another metabolic pathway included hydroxylation of either the benzyl ring or the methylene linker, also combined with *N*-demethylations or further hydroxylation (**A6**, **A7**, **A8**, **A12**, **A14**). The main hydroxylated phase I metabolites underwent further glucuronidation (**A15**, **A16**, **A17**, **A19**, **A20**) or sulphation (**A18**, **A21**).

**Figure 5:**
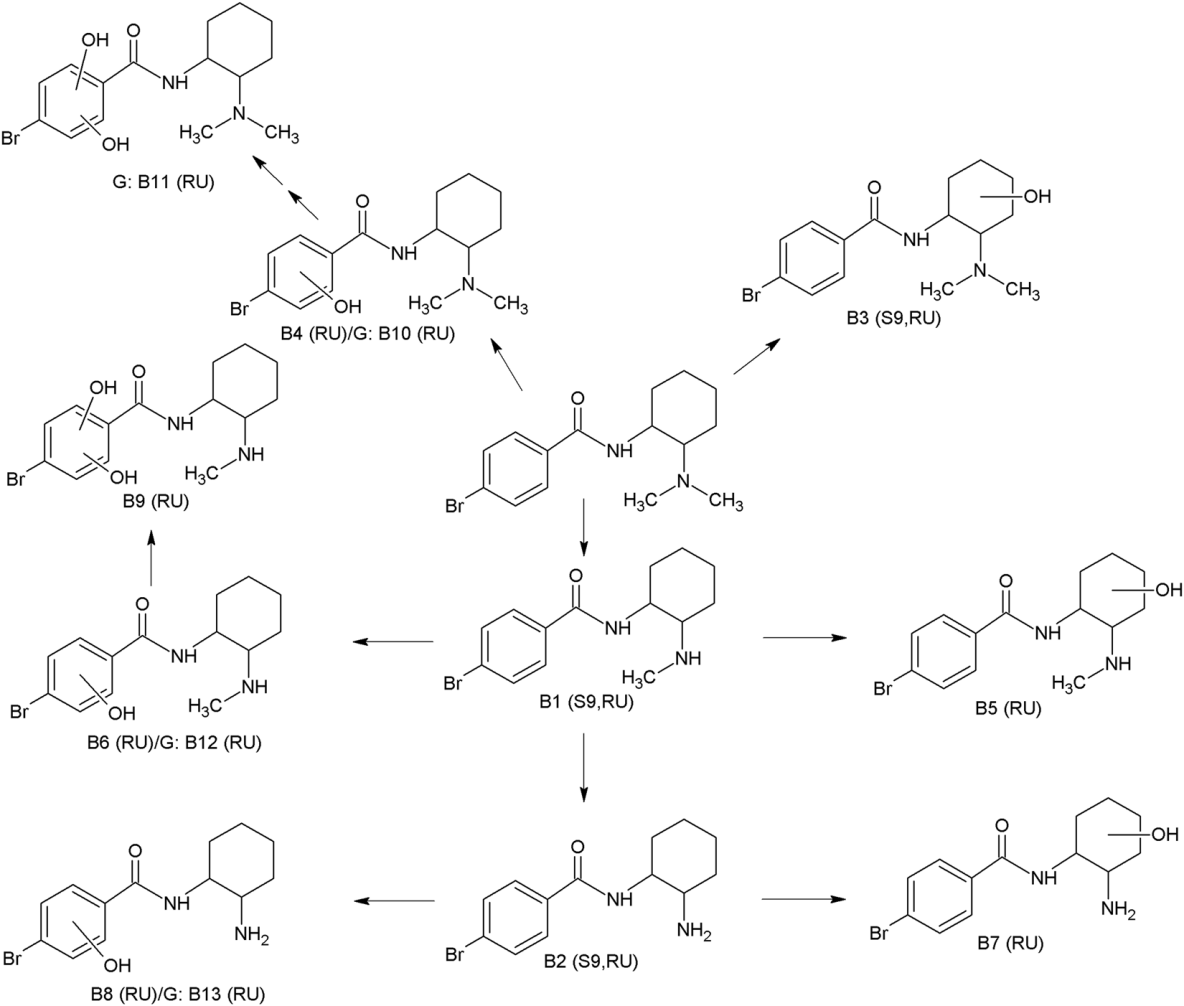
Metabolic pathways of U-47931E studied in rat urine (RU) and pooled human S9 fraction incubations (S9). Phase II metabolites: glucuronides (G). Numbering according to Table 2.

Figure 5 shows the proposed metabolic pathway of U-47931E. The main initial step in the metabolic pathways was *N*-demethylation (**B1**). Further metabolism steps were an additional *N*-demethylation (**B2**) or hydroxylation at the cyclohexyl ring (**B5**, **B7**) or the cyclophenyl ring (**B6**, **B8**). The *N*-demethylated hydroxy metabolite with hydroxylation at the aromatic system (**B6**) could be further hydroxylated (**B9**). Hydroxylation of the parent compound could occur at either the cyclohexyl (**B3**) or the benzyl ring (**B4**), forming two different metabolic pathways. All phase I metabolites with hydroxylation at the benzyl ring were conjugated to glucuronic acid (**B10**, **B11**, **B12**, **B13**), except for the *N*-demethylated dihydroxylated metabolite. Interestingly, no conjugates were formed after hydroxylation at the cyclohexyl ring even if higher amounts of corresponding phase I metabolites were found. Contrary to U-51754, no sulphated metabolites could be observed.

**Figure 6:**
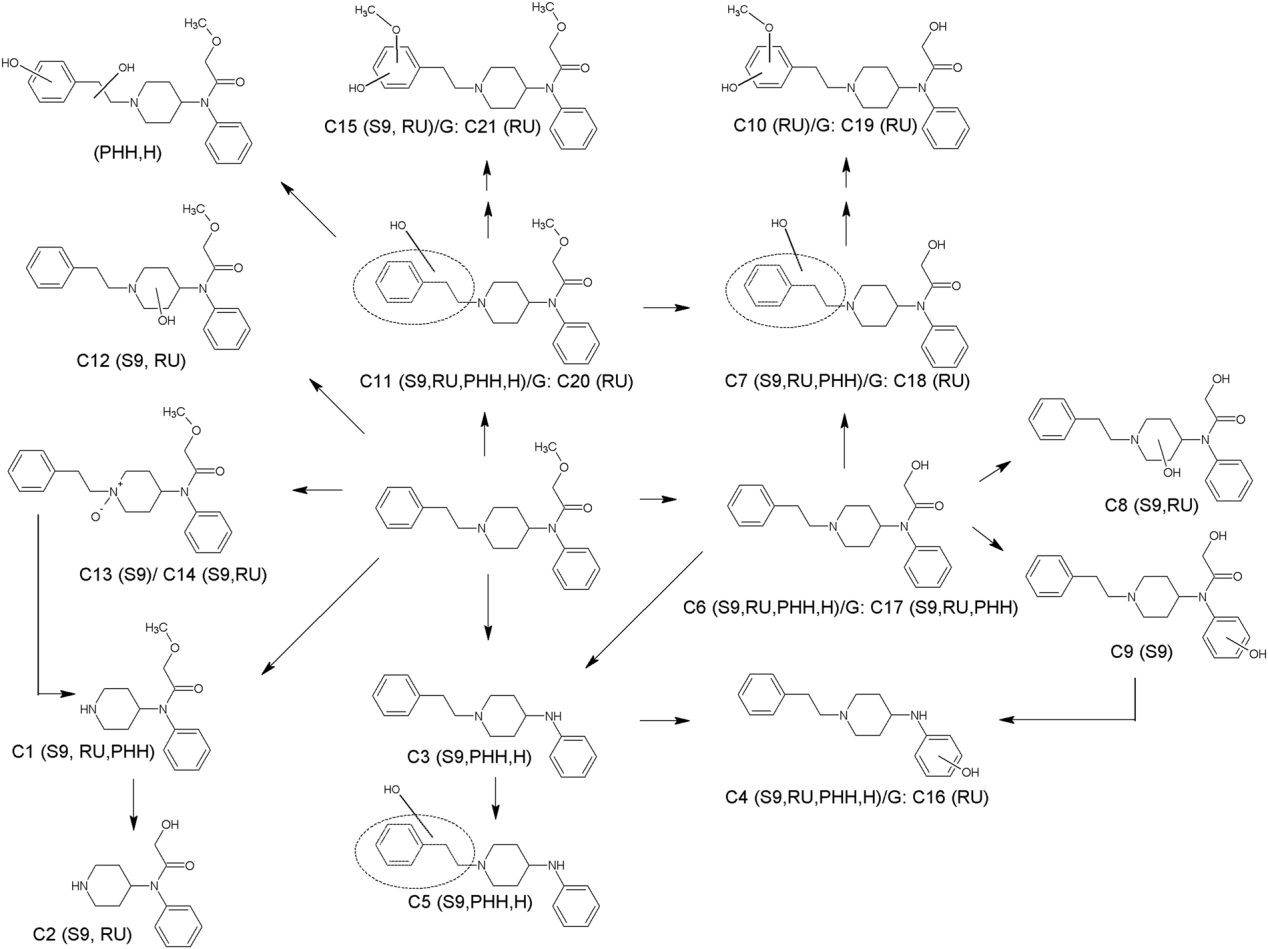
Metabolic pathways of methoxyacetylfentanyl studied in rat urine (RU), pooled human S9 fraction incubations (S9), pooled human hepatocytes (PHH)^18^ and/or various biological human samples (H)^18^. Phase II metabolites: glucuronides (G). Numbering according to Table 3.

Figure 6 shows the proposed metabolic pathways for methoxyacetylfentanyl. Five initial metabolic steps could be postulated. One metabolic pathway was the *N*-dealkylation (**C1**). After *N*-dealkylation, the nor-metabolite was *O*-demethylated further (**C2**). On the other hand, one initial metabolic step was the *O*-demethylation (**C6**) itself, which was followed by further alkyl, piperidine or aryl hydroxylation (**C7**, **C8**, **C9**) or underwent methylation after two-fold hydroxylation (**C10**). The third metabolic pathway included the amide hydrolysis to the 4-ANPP derivative (**C3**) followed by hydroxylation (**C4**, **C5**). One additional metabolic pathway was the hydroxylation of the parent compound at either the alkyl moiety (**C11**) or the piperidine ring (**C12**) followed by methylation after two-fold hydroxylation (**C15**). Furthermore, hydroxylation to the respective *N*-oxide (**C13**, **C14**) could be observed. Some hydroxylated metabolites were further conjugated to glucuronic acid (**C16**, **C17**, **C18**, **C19**, **C20**, **C21**), whereas sulphates could not be detected.

## Discussion

The parent compounds U-51754 and U-47931E as well as methoxyacetylfentanyl showed only very low signal abundances in rat urine. Concerning other U-substances, comparable results were found in previous studies^33^. In the case of fentanyl, less than 8% is excreted unchanged and approximately 85% is eliminated metabolised in faeces and urine within 72 h^34^. It can be assumed that fentanyl analogues will show a comparable behaviour. Following the administration of isofentanyl and 3-methylfentanyl, Meyer *et al*. could not detect the parent drugs in urine either^30^. In other urinary metabolism studies, the parent compounds could be detected with high abundances^35,36^. However, since urinary excretion of parent compounds predominantly occurs via metabolites, it is essential to determine metabolites that can serve as targets for urine screening.

After administration of U-51754, the *N*-demethyl-hydroxy metabolite was the most abundant one in the urine specimens, which is a rough assessment considering possible differences in abundance in different MS types. In addition, the *N*,*N*-bisdemethylated and hydroxylated metabolite were abundant, likewise the monohydroxylated metabolite and *N*-demethyl-U-51754. All these metabolites showed a distinctively higher abundance compared with the parent compound. In line with our results, the *N*-demethylated metabolite provided abundant signal intensities for other U-substances as well in previous studies and, as a consequence, should be used as a target^28,37^. However, even the *N*-demethyl-hydroxy metabolite and the hydroxy metabolite might be suitable as targets. In contrast, conjugated metabolites only showed low signal abundance and could be excluded.

For U-47931E, the most abundant metabolites were the one- and two-fold *N*-demethylated metabolites with or without further hydroxylation and the hydroxy metabolite. However, all metabolites exhibited signal intensities lower than that of the parent compound. Nonetheless, the *N*-demethylated metabolite reached good detectability in human *in vitro* assays as well and might be preferentially used as an additional urinary marker. Conjugated metabolites were the least abundant metabolites and thus excluded as possible targets.

The alkyl hydroxy metabolite was the most abundant metabolite in rat urine samples after administration of methoxyacetylfentanyl, showing a much higher signal abundance than that of the parent compound. Hydroxy metabolites were found to be one of the main metabolites of different fentanyl analogues in other studies as well, but not yet for methoxyacetylfentanyl^18,33^. Furthermore, the nor-metabolite and the *O*-demethylated metabolite were also abundant metabolites. In line with these results, the nor-metabolite has already been described as the main metabolite of many fentanyl analogs^31,34^. As far as humans are concerned, Mardal *et al*. observed the parent compound, together with the *O*-demethylated metabolite and the amide hydrolysis product 4-ANPP, as representing the highest signal intensities in different biological samples^18^. However, this result was obtained by studying different tissue and blood samples and only one urine sample. This fact could have affected the detectability of different metabolites, as in our study. Furthermore, 4-ANPP was found to be a minor metabolite in other metabolism studies for other fentanyls^38–40^. Taking these findings together, the nor-metabolite and the *O*-demethylated metabolite might be the most suitable targets for urine screening approaches. Supporting this, the *O*-demethylated metabolite was also elucidated as the main metabolite for the structurally related fentanyl analogue Ocfentanil^41^. Conjugated metabolites showed only low signal abundance, turning out to be neglected as possible targets.

Owing to the lack of authentic human urine samples, incubations with phS9 incubations were performed in this study and compared with the metabolites formed by rats. Incubations with phS9 fraction are one alternative model to HLM or hepatocyte cell cultures for the assessment of toxicokinetic data.^1,21,31,42^. However, *in vitro* models are limited, running the risk of missing metabolites. As a comprehensive model, an animal^25^ might be used, but the potential of interspecies differences in enzyme activity has to be considered^43,44^. Well-established models for metabolism studies are rats. In accordance with previous studies, this animal model can be used for urinary identification of metabolites after oral administration and thus was used in this study^23,24,45^. A comparison of all metabolites formed *in vivo* and *in vitro* is shown in Table 4. In general, major phS9 metabolites were in good agreement with major rat urine metabolites for all NSOs tested and the same biotransformations were observed. Many deviations between phS9 and urine metabolites can be explained by the different time allowed for metabolism and the missing recirculation of metabolites *in vitro* compared with *in vivo*. This leads to fewer metabolites *in vitro* in general and a lower prevalence of second- or third-generation metabolites. This is in accordance with previous studies comparing *in vitro* and *in vivo* metabolism using rat urine and phS9^22^. The low formation of phase II metabolites in phS9 incubations might be caused by the low formation of underlying phase I metabolites.

**Table 4:** Phase I and II metabolites of U-51754, U-47931E, and methoxyacetylfentanyl found *in vitro* (phS9 fractions) and *in vivo* (rat urine) compared to those detected in pooled human hepatocytes (PHH) and various biological human samples published by Mardal *et al*.^18^.

ID	Analyte	phS9	Rat urine	PHH	Human samples
**U-51754**					
A1	*N*-demethyl I-	+	+		
A2	*N*-demethyl II-	+			
A3	*N*,*N*-bisdemethyl-	+			
A4	*N*,*N*,*N*-tridemethyl-	+			
A5	Hydroxy I-	+			
A6	Hydroxy II-	+	+		
A7	Dihydroxy I-		+		
A8	Dihydroxy II-		+		
A9	*N*-oxide-	+			
A10	*N*-oxide-hydroxy-	+			
A11	*N*-demethyl-hydroxy I-	+	+		
A12	*N*-demethyl-hydroxy II-		+		
A13	*N*,*N*-bisdemethyl-hydroxy I-	+			
A14	*N*,*N*-bisdemethyl-hydroxy II-		+		
A15	hydroxy-glucuronide		+		
A16	dihydroxy-glucuronide		+		
A17	*N*-demethyl-hydroxy-glucuronide		+		
A18	*N*-demethyl-hydroxy-sulfate		+		
A19	*N*-demethyl-dihydroxy-glucuronide		+		
A20	*N*,*N*-bisdemethyl-hydroxy-glucuronide		+		
A21	*N*,*N*-bisdemethyl-hydroxy-sulfate		+		
**U-47931E**					
B1	*N*-demethyl-	+	+		
B2	*N*,*N*-bisdemethyl-	+	+		
B3	Hydroxy I-	+	+		
B4	Hydroxy II-		+		
B5	*N*-demethyl-hydroxy I-		+		
B6	*N*-demethyl-hydroxy II-		+		
B7	*N*,*N*-bisdemethyl-hydroxy I-		+		
B8	*N*,*N*-bisdemethyl-hydroxy II-		+		
B9	*N*-demethyl-dihydroxy-		+		
B10	Hydroxy-glucuronide		+		
B11	Dihydroxy-glucuronide		+		
B12	*N*-demethyl-hydroxy-glucuronide		+		
B13	*N*,*N*-bisdemethyl-hydroxy-glucuronide		+		
**Methoxyacetylfentanyl**					
C1	*N*-dealkyl- (Nor)	+	+	+	
C2	*N*-dealkyl-*O*-demethyl-	+	+		
C3	Amide hydrolyzed-	+		+	+
C4	Amide hydrolyzed hydroxy I-	+	+	+	+
C5	Amide hydrolyzed hydroxy II-	+		+	+
C6	*O*-demethyl-	+	+	+	+
C7	*O*-demethyl-hydroxy I-	+	+	+	
C8	*O*-demethyl-hydroxy II-	+	+		
C9	*O*-demethyl-hydroxy III-	+			
C10	*O*-demethyl-hydroxy-methoxy-		+		
C11	Hydroxy I-	+	+	+	+
C12	Hydroxy II-	+	+		
C13	*N*-oxide I-	+			
C14	*N*-oxide II-	+	+		
C15	Hydroxy-methoxy-	+	+		
C16	Amide hydrolysed hydroxy-glucuronide		+		
C17	*O*-demethyl-glucuronide	+	+	+	
C18	*O*-demethyl-hydroxy-glucuronide		+		
C19	*O*-demethyl-hydroxy-methoxy-glucuronide		+		
C20	Hydroxy-glucuronide		+		
C21	Hydroxy-methoxy-glucuronide		+		

For U-51754, seven out of 14 phase I metabolites could be detected in rat urine and 10 out of 14 in phS9 incubations. Only three metabolites were identical in both models. The different sites of hydroxylation were one of the main differences between both models, but the main metabolic steps such as single *N*-demethylation, hydroxylation, and single *N*-demethylation combined with hydroxylation were comparable for both models. As already mentioned, different hydroxylation sites might be a species-related difference. Phase II metabolites were only detected in rat urine.

In summary, for U-47931E, more metabolites could be detected *in vivo* than *in vitro*. Only three out of nine metabolites were found in phS9 incubates. In particular, the formation of multiple-step metabolites was more dominant in the *in vivo* system. Phase II metabolites were again only found *in vivo*. However, the most abundant metabolites were comparable in both models.

For methoxyacetylfentanyl, eleven out of 15 phase I metabolites were found in rat urine and 14 out of 15 in phS9. Ten metabolites were identical in both models. The alkyl hydroxylated 4-ANPP could only be observed in phS9 incubations as well as the *O*-demethylated metabolite with further aryl hydroxylation. In rat urine, six phase II metabolites were found, whereas in phS9 incubations only one glucuronide could be identified. This is in accordance with findings published by Richter *et al*.^21^. In summary, most metabolic steps, particularly the main reactions such as the *N*-dealkylation, *O*-demethylation, and hydroxylation, were comparable in both models.

In general, the results of this study are in good agreement with those concerning comparable compounds. Owing to the lack of data from further metabolism studies on U-51754 or U-47931E, the results of this study must be compared with data from U-47700 or other U-substances such as U-49900. In terms of *N*-demethylation as a major metabolic pathway for U-51754 and U-47931E, our results are in good agreement with data already published on the metabolism of U-47700 in human liver microsomes (HLM) and human case samples^28,37,46,47^. Compared with previous studies^33,47^, this is the first study detecting phase II metabolites in this class of substances. Only for AH-7921, a structurally related compound, a glucuronide has already been described^48^. Contrary to U-51754, no sulphated metabolites could be observed for U-47931E, but the main phase I pathways determined for this substance were in accordance with our results for U-51754 or further studies on other U-substances^33^. In total, fewer metabolites of U-47931E were formed in comparison to U-51754.

In general, the findings concerning the formation of metabolites of methoxyacetylfentanyl showed, on the one hand, similarities to other fentanyl-analogue metabolism studies^1,18^, but, on the other, differences regarding the formation of dihydroxylated metabolites. *N*-dealkylation was already found to be a common metabolic reaction for other fentanyls^49,50^. Further, the formation of 4-ANPP has already been described as a metabolic pathway for many other fentanyl analogues^51–53^. Other initial metabolic steps such as *O*-demethylation and hydroxylation have been published for methoxyacetylfentanyl by Mardal *et al*. or in previous studies of other fentanyls as well^18,31^. However, Mardal *et al*. did not detect *N*-oxidation. In contrast, they found the dihydroxylated metabolite. Furthermore, they could distinguish between hydroxylation at the ethyl side chain and the phenyl ring of this moiety by means of mass spectrometry. These differences could be an effect of different settings of the mass spectrometry analysis used in that study.

## Conclusions

The major phase I and II metabolites of the three NSOs, U-51754, U-47931E, and methoxyacetylfentanyl, were tentatively identified in phS9 as well as in rat urine specimens after oral administration. Concerning U-51754 and U-47931E, *N*-demethylation of the amine moiety and hydroxylation of the hexyl ring as well as combinations thereof led to the most abundant metabolites. *N*-dealkylation to the nor-metabolite, *O*-demethylation, and hydroxylation at the alkyl part of the molecule were observed to be the most abundant metabolites of methoxyacetylfentanyl. These findings indicate that metabolites are essential urinary targets for detecting these NSOs and confirm their consumption, as most of the parent compounds could only be detected with minor abundance in rat urine. In general, the results of this study are in good agreement with those concerning comparable compounds obtained from HLM incubations, human hepatocytes, and/or human cases.

### Compliance with ethical standards

The authors declare that the experiments have been conducted in accordance with all applicable institutional, national, or international guidelines for care and use of rats.

## Supplementary information

Electronic Supplementary Material

## Data Availability

Datasets generated during and/or analyzed during the current study are not publicly available but are available from the corresponding author on reasonable request.
