# Supplementary material for: Studies on the in vitro and in vivo metabolism of the synthetic opioids U-51754, U-47931E, and methoxyacetylfentanyl using hyphenated high-resolution mass spectrometry
Source: Sci Rep. 2019 Sep 24;9:13774. doi: 10.1038/s41598-019-50196-y (PMC6760207; doi:10.1038/s41598-019-50196-y)
Supplement: Supplementary file 1 — Electronic Supplementary Material [file 41598_2019_50196_MOESM1_ESM.pdf]

## Electronic Supplementary Material

# Studies on the in vitro and in vivo metabolism of the synthetic opioids U-51754, U-47931E, and methoxyacetylfentanyl using hyphenated high-resolution mass spectrometry

Frederike Nordmeier[1], Lilian H. J. Richter[2], Peter H. Schmidt[1], Nadine Schaefer[1], and Markus R. Meyer[2,*]

[1]Institute of Legal Medicine, Saarland University, 66421 Homburg, Germany
[2]Department of Experimental and Clinical Toxicology, Institute of Experimental and Clinical Pharmacology and Toxicology, Saarland University, 66421 Homburg, Germany

**Fig. S1.** HR-MS/MS spectra of selected phase I and II metabolites of U-51754, U-47931E, and methoxyacetylfentanyl. The spectra with proposed structures, retention times (RT), detected in rat urine (RU) and/or pooled human S9 fraction incubations (S9), and predominant fragmentation patterns of the metabolites are arranged according to their presentation in the text.

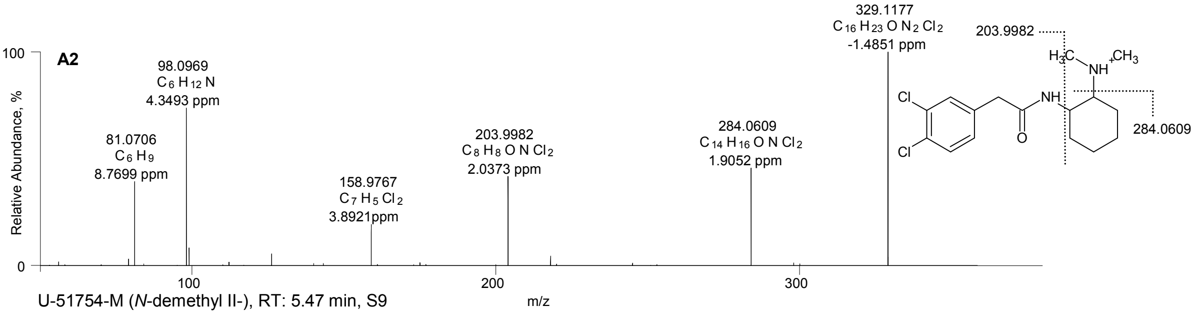


U-51754-M (*N*-demethyl II-), RT: 5.47 min, S9

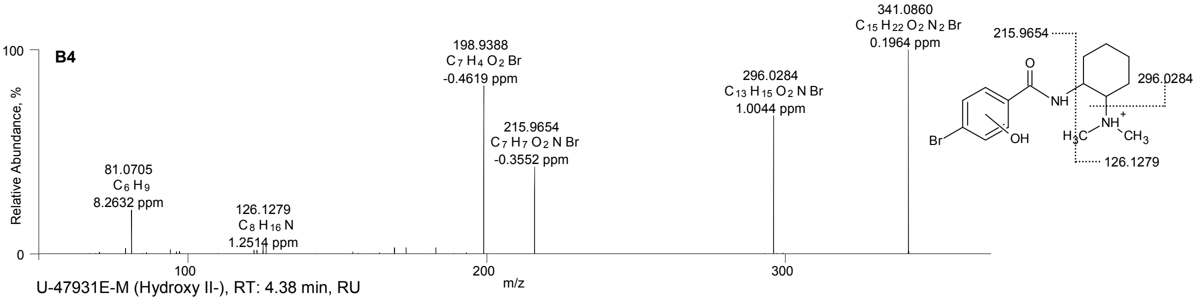


U-47931E-M (Hydroxy II-), RT: 4.38 min, RU

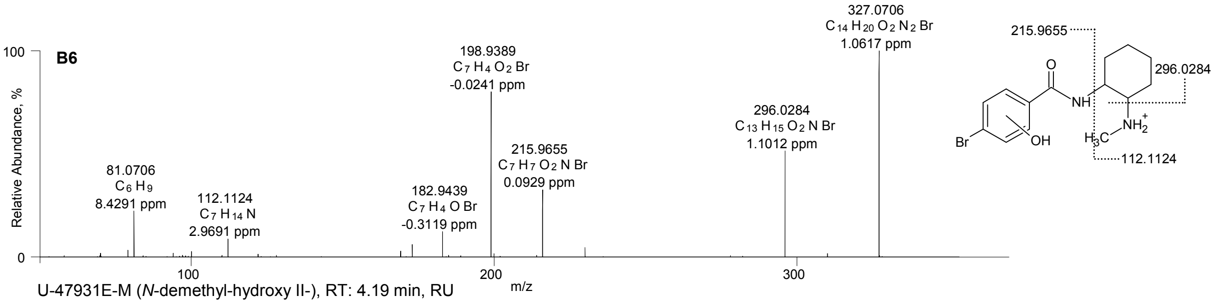


U-47931E-M (*N*-demethyl-hydroxy II-), RT: 4.19 min, RU

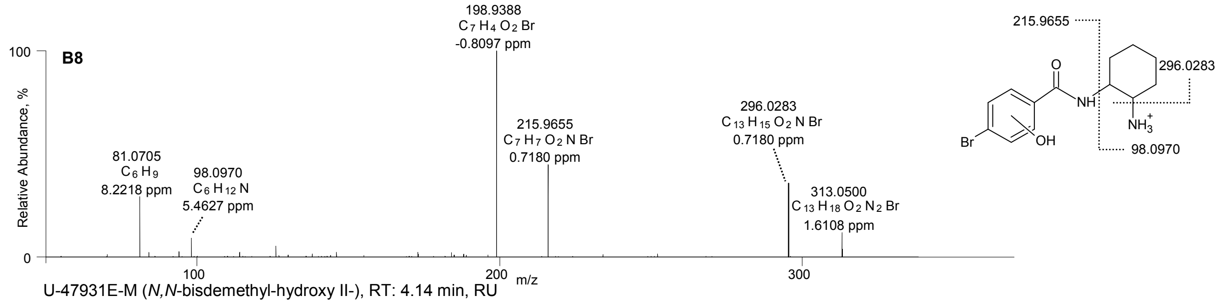


U-47931E-M (*N*,*N*-bisdemethyl-hydroxy II-), RT: 4.14 min, RU

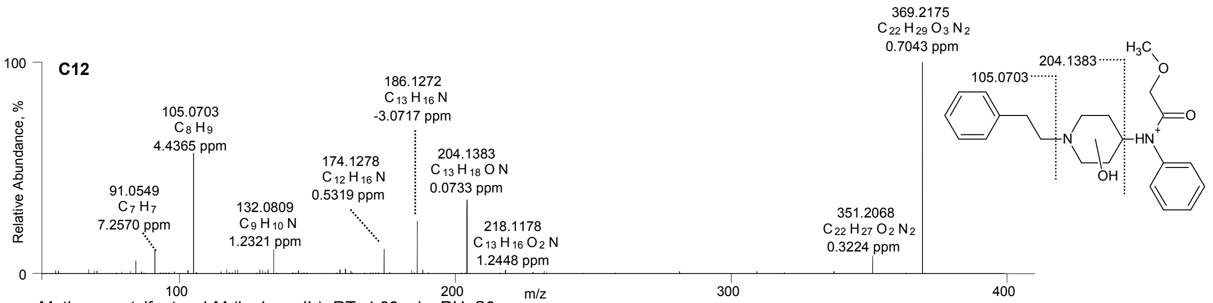


Methoxyacetylfentanyl-M (hydroxy II-), RT: 4.38 min, RU, S9

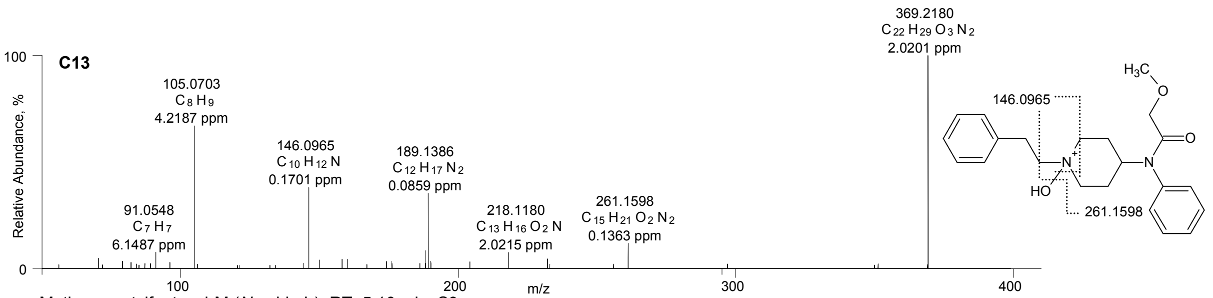


Methoxyacetylfentanyl-M (*N*-oxide I-), RT: 5.10 min, S9

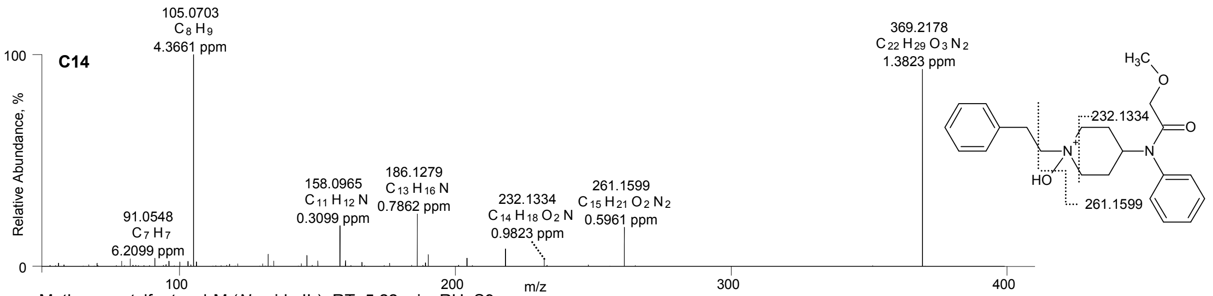


Methoxyacetylfentanyl-M (*N*-oxide II-), RT: 5.22 min, RU, S9

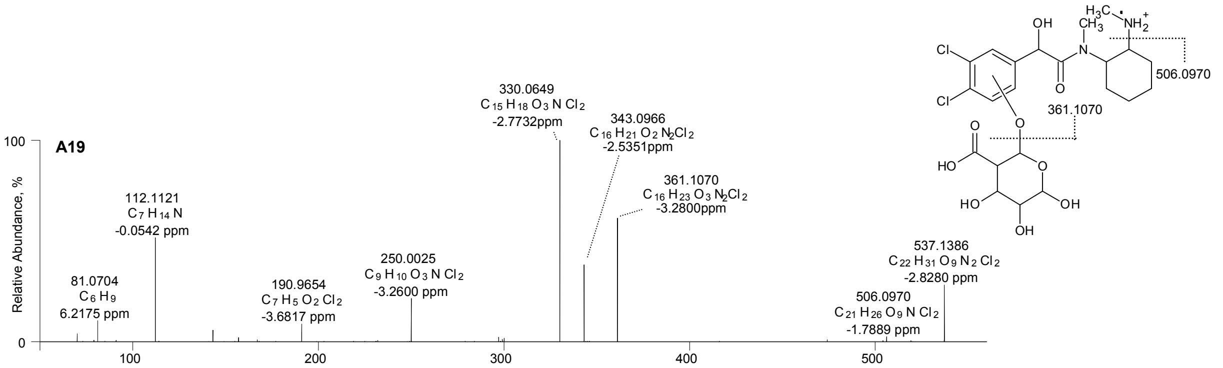


U-51754-M (*N*-demethyl-dihydroxy-glu), RT: 3.82 min, RU
m/z

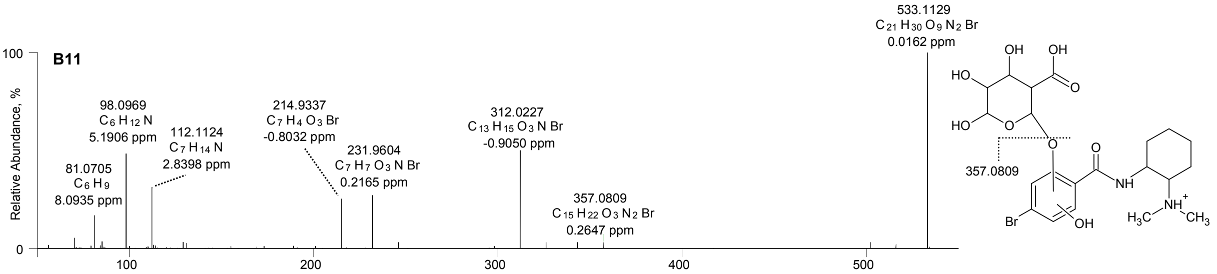


U-47931E-M (Dihydroxy-glu), RT: 4.35 min, RU

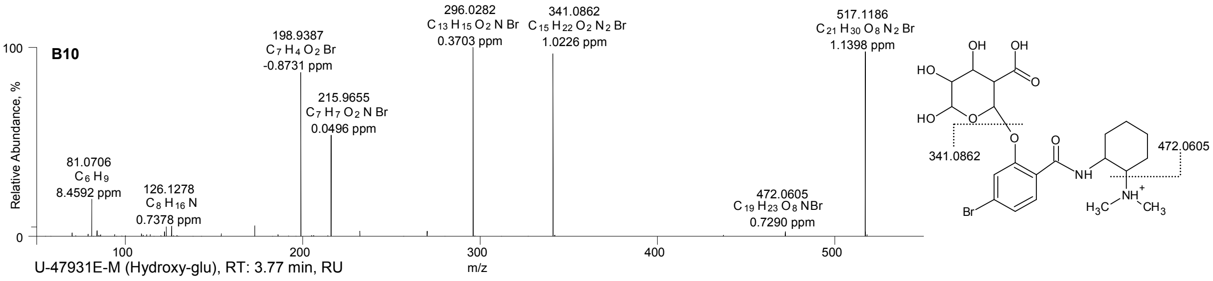


U-47931E-M (Hydroxy-glu), RT: 3.77 min, RU

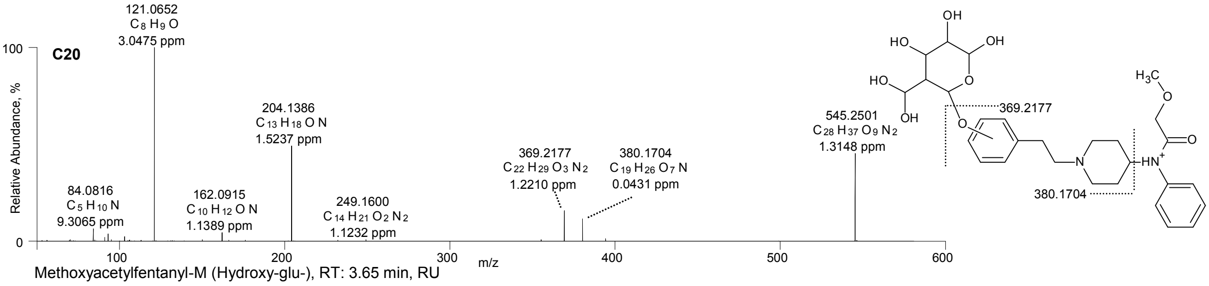


Methoxyacetylfentanyl-M (Hydroxy-glu-), RT: 3.65 min, RU

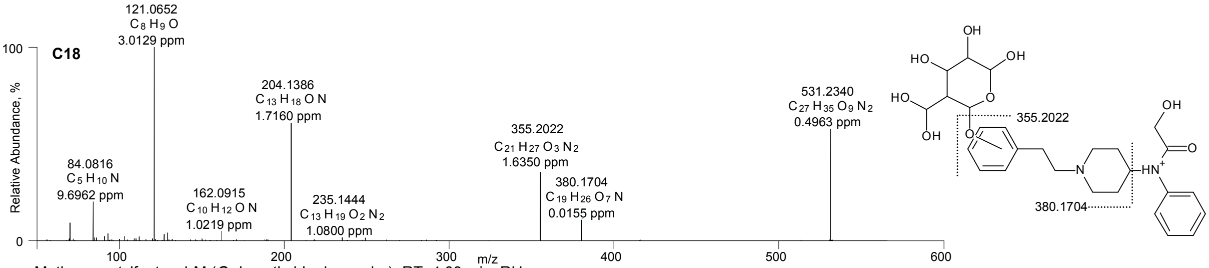


Methoxyacetylfentanyl-M (*O*-demethyl-hydroxy-glu-), RT: 4.03 min, RU

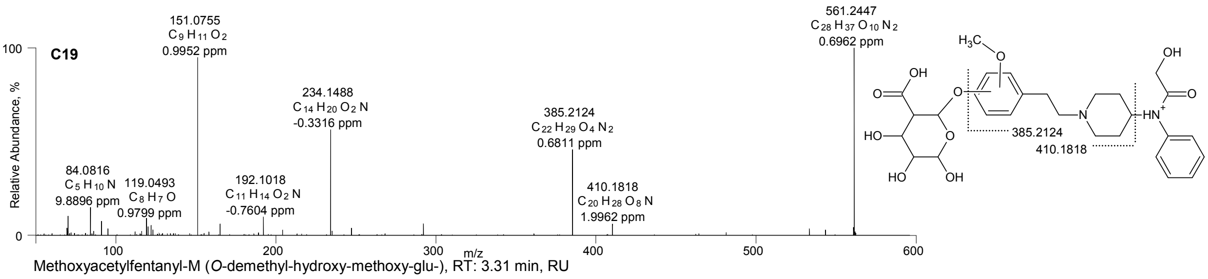


Methoxyacetylfentanyl-M (*O*-demethyl-hydroxy-methoxy-glu-), RT: 3.31 min, RU
